# Using recombinant Rho protein antagonist in acute spinal cord injury; does this go further from conventional decompressions?

**DOI:** 10.3389/fneur.2013.00005

**Published:** 2013-02-05

**Authors:** Nima Hafezi-Nejad, Vafa Rahimi-Movaghar

**Affiliations:** ^1^Department of Neurosurgery, Sina Trauma and Surgery Research Center, Tehran University of Medical SciencesTehran, Iran; ^2^Student's Scientific Research Center, Tehran University of Medical SciencesTehran, Iran

New evidence has suggested the Rho signaling pathway as a target for treatment in acute spinal cord injury (SCI). The Rho signaling pathway plays an important role in neuronal growth inhibition after a CNS injury. Block of Rho pathway is a new potential method to improve recovery after an SCI. Other studies by our group had further revealed the underlying mechanisms of this pathway (Firouzi et al., 2011).

BA-210 (Trademarked as Cethrin) is a newly designed Rho pathway antagonist. A “Phase I/IIa Clinical Trial of a Recombinant Rho Protein antagonist in Acute Spinal Cord Injury” by Fehlings et al. (2011) has been recently published to evaluate BA-210 specifically in course of SCI. Implications of the findings of this study can potentially change the management of this condition around the world. However, we would like to highlight some concerns.

The article referenced two guideline reviews on SCI and its spontaneous recovery (Fawcett et al., 2007; Steeves et al., 2007). They reported the results of recovery after the application of BA-210 as well. This may cause misinterpretation when comparing their results with the mentioned guidelines. Guidelines were reviews of investigations including those by Kirshblum et al. (2004), Waters et al. (1993), Geisler et al. (2001) and European Multicenter study in Spinal Cord Injury (EMSCI). The first observations to note are the differences between the study groups. Excluding gunshots, stab wounds, and transecting injuries in the current study, leaves the only those patients who are more likely to recover. The same occurs in excluding patients who are more likely to die in the following 6 months as well as conditions that make patients unable or unwilling to participate. Their criteria for excluding selected patients raise the concern that the study group was more likely to recover spontaneously. Neither of the above mentioned conditions was assigned in the compared studies.

The current study reported 31% (5/16) recovery from ASIA A to at least ASIA C at cervical level of injury along with 6.3% (2/32) recovery at thoracic level. They encountered an overall of recovery of around 14.6% (7/48). The authors considered this to be encouraging. However, we compared this to a meta-analysis by La Rosa et al. They analyzed the summation of at least 10 series of reports on patients with complete SCI. Early decompression was matched to the timing of surgery in current study. In all, 42% (50/119) of the patients were considered neurologically improved. Improvement was defined by at least one grade progress according to the Frankel's scale; including both cervical and thoracic levels of injury. Although 42% recovery in La Rosa et al.'s meta-analysis regards to advances from ASIA A to B, it is still far behind 14.6%. This can be a proper baseline and reference for comparison; beside the 10% predicted rate of conversion derived for untreated cervical SCI in the Fehlings et al.'s article (La Rosa et al., 2004). Other studies insist on the low rate of recovery after thoracic SCI. However, T12 injuries are similar in nature to L1 injuries rather than thoracic level injuries. Separations of injuries on T12 are still recommended to avoid over-reporting in recoveries of thoracic SCI (Rahimi-Movaghar, 2005; Rahimi-Movaghar et al., 2006).

Fehlings et al. (2011) did not adjust the results for dural tears. Dural tears were reported in 31% of the patients. This can be of importance in assessing Cethrin's tolerability and side effects. Moreover, it can potentially affect the neurological outcome after drug administration. Presence of dural tears leaves the possibility of confounding effects intact.

Finally, can we expect a single application of an agent in a proximate time to the injury to declare further trend of “linear recovery” in its following 12 months? Further investigations as well as phase II and III clinical trials can answer this question. Revealing the actual efficacy of BA-210, particularly by a more inclusive study group and an adjusted comparison is needed.
